# Comparative Studies on Cellular Behaviour of Carnation (*Dianthus caryophyllus* Linn. cv. Grenadin) Grown *In Vivo* and *In Vitro* for Early Detection of Somaclonal Variation

**DOI:** 10.1155/2013/686752

**Published:** 2013-05-22

**Authors:** Jamilah Syafawati Yaacob, Rosna Mat Taha, Arash Khorasani Esmaeili

**Affiliations:** Institute of Biological Sciences, Faculty of Science, University of Malaya, 50603 Kuala Lumpur, Malaysia

## Abstract

The present study deals with the cytological investigations on the meristematic root cells of carnation (*Dianthus caryophyllus* Linn.) grown *in vivo* and *in vitro*. Cellular parameters including the mitotic index (MI), chromosome count, ploidy level (nuclear DNA content), mean cell and nuclear areas, and cell doubling time (Cdt) were determined from the 2 mm root tip segments of this species. The MI value decreased when cells were transferred from *in vivo* to *in vitro* conditions, perhaps due to early adaptations of the cells to the *in vitro* environment. The mean chromosome number was generally stable (2*n* = 2*x* = 30) throughout the 6-month culture period, indicating no occurrence of early somaclonal variation. Following the transfer to the *in vitro* environment, a significant increase was recorded for mean cell and nuclear areas, from 26.59 ± 0.09 **μ**m^2^ to 35.66 ± 0.10 **μ**m^2^ and 142.90 ± 0.59 **μ**m^2^ to 165.05 ± 0.58 **μ**m^2^, respectively. However, the mean cell and nuclear areas of *in vitro* grown *D. caryophyllus* were unstable and fluctuated throughout the tissue culture period, possibly due to organogenesis or rhizogenesis. Ploidy level analysis revealed that *D. caryophyllus* root cells contained high percentage of polyploid cells when grown *in vivo* and maintained high throughout the 6-month culture period.

## 1. Introduction

Carnation or *Dianthus caryophyllus* is a herbaceous perennial plant that can grow up to 80 cm tall, with grayish green or blue-green glaucous leaves. Carnation flowers are sweetly scented, about 3–5 cm in size (diameter), and are either produced singly or in a bunch. Carnation flowers are naturally bright pinkish-purple in colour, but other colourful cultivars of this plant had been developed such as carnations with white, red, green, and yellow flowers. The increasing demand for carnations has rendered this species to be a special candidate for mass propagation through tissue culture. Frey and Janick [1] reported on organogenesis observed from carnation petals cultured on MS [2] medium supplemented with 0.05 *μ*M TDZ and 0.5 *μ*M NAA. Ali et al. [3] showed *in vitro* shoot formation from apical and nodal meristems of carnation when cultured on an MS medium fortified with BAP. On the other hand, Watad et al. [4] observed *in vitro* shoot formation from internode explants cultured on an MS medium supplemented with TDZ and NAA. However, the effect of plant growth regulators, following the transfer of *D. caryophyllus* cells from *in vivo* to *in vitro* conditions and investigation at the cellular level, had not been reported before.

According to Karp [5], basic cytological technique is very important and useful for the determination of accurate chromosome number and structure and should be in routine use for regenerated plants in tissue culture. Cytological studies, that is, measurements of the mitotic index (MI), mean cell and nuclear areas, chromosome count, and cell doubling time (Cdt), were carried out to elucidate any differences or changes that occurred in *in vivo* and *in vitro* grown *D. caryophyllus* plants at the cellular level, enabling the easy and early detection of somaclonal variations. Cytology facilitates chromosomal and cell division studies in plants [6] as well as enables the detection of embryogenic callus from nonembryogenic callus [7] and *in vitro* flowering [8, 9]. Thus, the aims of the present study are to compare at the cellular level the characteristics of tissues obtained from *in vivo* and *in vitro* environments, to detect any cellular changes when cells are transferred from the *in vivo* to the *in vitro* system, and to detect any occurrence of somaclonal variation at the cellular level.

## 2. Materials and Methods

### 2.1. Sterilization of Seeds and Determination of Standard Growth of Primary Roots

One hundred seeds of *Dianthus caryophyllus* Linn. cv. Grenadin bought from Yates Company, Australia, were surface-sterilized following standard tissue culture protocols [10] but with minor modifications. The seeds were washed using sterile distilled water, followed by treatments with 100%, 70%, and 30% (v/v) commercial bleach (Clorox) for 2 minutes at each concentration. Two drops of Tween-20 were also added during the treatment with 100% (v/v) Clorox to facilitate the sterilization process and reduce surface tension. The seeds were rinsed with sterile distilled water to remove excess Clorox, submerged in 70% (v/v) ethanol, and finally rinsed 3 times with sterile distilled water.

The seeds were then germinated on preautoclaved moist cotton wool and maintained in the culture room at 25  ±  1°C with 16 hours light and 8 hours dark for 5 days. The growth of primary roots was monitored on a daily basis, whereby the length of the primary roots was measured at the same time every day to determine the standard growth of *Dianthus caryophyllus* primary roots. A graph of primary root length against time was plotted and linear regression was obtained, yielding the optimum root length (standard) to be used in subsequent cytological experiments.

### 2.2. Plantlet Regeneration and Determination of Optimum Rooting Media

The seeds of *Dianthus caryophyllus* Linn. cv. Grenadin bought from Yates Company, Australia, were surface-sterilized and germinated on moist cotton wool as previously described. Four-day-old primary roots with a standard length of 11.15  ±  0.33 mm were used to initiate the cultures of this species. The 4-day-old primary roots were excised and immersed in 70% (v/v) ethanol for a few seconds, followed by washing 3 times with sterile distilled water prior to tissue culture initiation. The primary root segments were cultured on MS [2] media supplemented with various combinations and concentrations of plant hormones, such as 0.5–3.0 mg L^−1^ *α*-naphthalene acetic acid (NAA) and 0.5–3.0 mg L^−1^ 6-benzyl aminopurine (BAP). The media were added with 30 g/L sucrose, pH 5.8  ±  0.1, solidified with 8 g/L agar technical no. 4, and autoclaved at 120°C for 20 minutes. The cultures were maintained in the culture room at 25  ±  1°C with 16 hours light and 8 hours dark for 6 months.

### 2.3. Morphology of *Ex Vitro* and *In Vivo* Grown Plants

Complete *Dianthus caryophyllus* plantlets were transferred to covered vases containing a 1 : 1 : 1 mixture of sand : garden soil : burnt soil and acclimatized in the culture room at 25  ±  1°C with light intensity of 800–1100 lux and a photoperiod of 16 hours light and 8 hours dark for 4 weeks. The plants were watered twice daily with distilled water. The plantlets were subsequently transferred to a greenhouse at 18  ±  2°C with light intensity of 400–1200 lux and a photoperiod of 12 hours light and 12 hours dark, and their growth performance in the natural environment was monitored. The morphological features such as the shape of the leaves, flowers, plant height, and mean leaf diameter of both *in vivo* and *ex vitro Dianthus caryophyllus* were compared to determine any morphological irregularities that might arise due to tissue culture stress or protocols.

### 2.4. Cytological Analysis on Roots of *In Vivo* and *In Vitro* Grown Plants

MS media supplemented with 2 mg L^−1^ NAA were found to be the most optimum media for the induction of roots of *Dianthus caryophyllus*; therefore primary roots obtained from *in vitro* cultures grown on this regeneration media were used throughout the experiment. Newly formed roots were excised from 1-, 2-, 3-, and 4-day-old; 1-, 2-, 3-, 4-, 5-, 6-, 7-, and 8-week-old; and 3-, 4-, and 6-month-old plantlets and preserved overnight in a 3 : 1 ratio of absolute alcohol : glacial acetic acid. The root segments were stained using Feulgen and made into permanent slides, prior to measurements of cellular parameters such as mitotic index (MI), chromosome number, DNA content and C value, mean nuclear and cell areas, and cell doubling time (Cdt) of this species.

The root segments were washed twice with distilled water for 5 minutes and then immersed in 5 M hydrochloric acid (HCl) for 20 minutes, followed by soaking in Feulgen for 2 hours. Feulgen-stained root tips without the root caps were transferred onto glass slides, and added with 1-2 drops of 45% (v/v) acetic acid. The slides were then made permanent based on the quick-freeze method described by Conger and Fairchild [11], and cover slides were mounted on the slides using DPX (Di-N-Butyl Phthalate in Xylene). Visualizations of the cells and chromosomes of *Dianthus caryophyllus* were conducted using a light microscope (Zeiss Axio Scope, Germany) connected to a Sony video camera, supported by VIDAS (Kontron Electronic, Germany).

Three permanent slides with at least 500 cells were observed to determine the mitotic index, which is the percentage of cells that are going through mitosis. The calculation of mitotic index was done based on the following formula:

(1) mitotic  index  (MI) =number  of  cells  undergoing  mitosistotal  number  of  cells×100,

whereby mitosis include cells in prophase, metaphase, anaphase and telophase.

Furthermore, at least 15 cells at metaphase spread were analyzed to determine the mean chromosome number of this species *in vivo* and *in vitro*. Cell doubling time (Cdt) of *in vitro* root meristem was measured from root cells with the highest mitotic index (MI), whereby the root segments were soaked in 0.5 mL of 0.025% (v/v) colchicine for 6 hours. Root segments previously soaked for 1, 2, 3, 4, 5, and 6 hours in colchicine were preserved in a 3 : 1 ratio of absolute alcohol : glacial acetic acid and made into permanent slides, as previously described.

**Figure 1:**
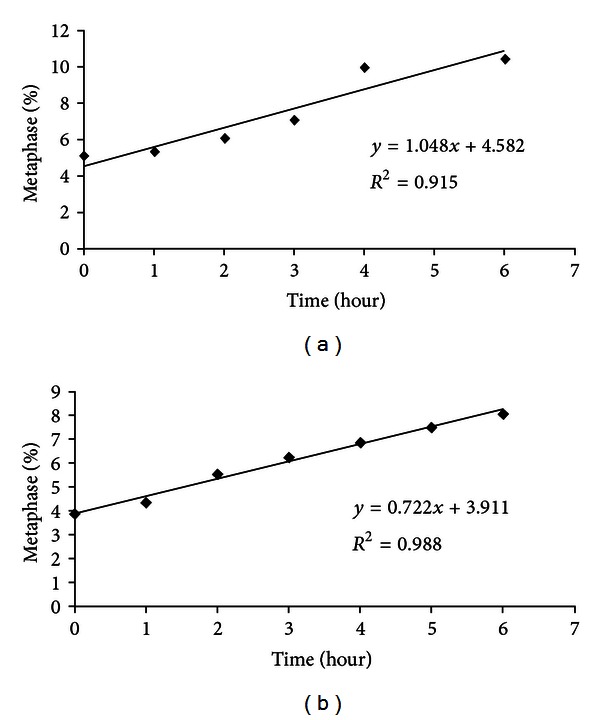
Relationship between percentage of metaphase frequency and duration of exposure to 0.025% colchicine for meristematic cells of *Dianthus caryophyllus* roots grown (a) *in vivo* and (b) on an MS medium supplemented with 2.0 mg L^−1^ NAA.

Four-day-old root segments of *in vivo* grown *Dianthus caryophyllus *with a standard length of 11.15  ±  0.33 mm (standard growth) were also subjected to cytological experiments. Cellular parameters such as mitotic index (MI), chromosome number, DNA content and C value, mean nuclear and cell areas, and cell doubling time (Cdt) of *in vivo* grown root meristem cells were measured and compared with those of *in vitro* grown plantlets. The Cdt was measured by plotting the graph of frequency of metaphase (depicted in terms of percentage) against time (duration of exposure to colchicine), which yielded linear regression lines (*y* = *mx* + *c*) for both *in vivo* and *in vitro *(Figure 1). The slope of the graph (*m*) represented the rate of accumulation of cells at the metaphase stage, which would be used in determining the cell doubling time (Cdt) based on Clowes' [12] formula:

(2) cell  doubling  time  (Cdt)=ln⁡⁡2m,

whereby *m* is the gradient of the graph.

### 2.5. Statistical Analysis

Different concentrations of hormones were assessed using randomized complete block design (RCBD) with 30 replicates to decrease error and enhance accuracy. Statistical analysis was conducted using statistical variance test (ANOVA) and compared using Duncan's multiple range test (DMRT) with the least significant differences at 5% level.

## 3. Results

### 3.1. Plantlet Regeneration and Determination of Optimum Rooting Media

In general, *in vitro* cultures of *D. caryophyllus* primary root segments (with a standard length of 11.15  ±  0.33 mm) on MS media supplemented with different combinations and concentrations of NAA and BAP were found to yield production of callus. White callus was formed when the root segments were cultured on MS media supplemented with 0.5–2.0 mg L^−1^ NAA and combinations of 0.5 mg L^−1^ NAA and 0.5, 1.0, and 2.0 mg L^−1^ BAP, 1.0 mg L^−1^ NAA and 0.5–1.5 mg L^−1^ BAP, 1.5 mg L^−1^ NAA and 2.0 mg L^−1^ BAP, and 2.0 mg L^−1^ NAA and 1.0 mg L^−1^ BAP (Table 1). White and green calluses were also produced from root segments cultured on MS media fortified with combinations of 1.5 mg/NAA and 0.5–1.5 mg L^−1^ BAP, 2.0 mg L^−1^ NAA and 0.5 mg L^−1^ BAP, and 2.0 mg L^−1^ NAA and 2.0 mg L^−1^ BAP (Table 1). On the other hand, additions of 2.0 mg L^−1^ NAA and 1.5 mg L^−1^ BAP yielded the formation of compact green callus (Table 1).

Direct root organogenesis was observed from cultures fortified with only BAP (0.5–2.0 mg L^−1^) and when high concentrations of NAA (3.0 mg L^−1^) were added (Table 1). Furthermore, indirect root organogenesis was also observed from the callus grown on MS media supplemented with NAA alone (0.5–2.0 mg L^−1^) and combinations of 0.5 mg L^−1^ NAA and 0.5 mg L^−1^ BAP, 1.0 mg L^−1^ NAA and 0.5 mg L^−1^ BAP, 1.5 mg L^−1^ NAA and 2.0 mg L^−1^ BAP, and 2.0 mg L^−1^ NAA and 1.0 mg L^−1^ BAP (Table 1). Production of roots was best achieved on MS supplemented with 2.0 mg L^−1^ NAA (Table 1), which yielded the highest number of roots (100%) and showed formation of secondary roots after as early as 7 days.

### 3.2. Cellular Behaviour Studies of *In Vivo* and *In Vitro* Grown Plants

Determination of standard growth of *D. caryophyllus* primary roots revealed that formation of primary roots was most optimum after 4 days, with a standard length of 11.15  ±  0.33 mm. The rate of root elongation (2.96 mm per day) was also determined from the standard growth graph, which yielded a linear regression line of *y* = 2.96*x* − 1.98 (data not shown). *In vivo* and *in vitro* grown *D. caryophyllus* root meristems with a standard length of 11.15  ±  0.33 mm were subjected to cytological analysis to determine their mean mitotic index (MI) values, mean chromosome numbers, mean nuclear and cell areas, DNA C values, and cell doubling time (Cdt). The effect of culture duration was also assessed in the current investigation.

It was observed that the mitotic index (MI) values decreased significantly when *in vivo D. caryophyllus *cells entered the tissue culture system, from 43.51  ±  2.14% (*in vivo*) to 41.20  ±  0.79%, 40.23  ±  1.30%, and 37.35  ±  0.40 after 1, 2, and 3 days in culture, respectively (Table 2). On the fourth day, the mitotic index was found to be astonishingly low (32.32  ±  1.55%) compared to that of the *in vivo* plant (Table 2). Interestingly, a significant increase in MI values was recorded after 1 and 3 weeks of culture, with MI values of 39.20  ±  1.54% and 43.77  ±  2.33%, respectively (Table 2). The MI values gradually decreased with increasing culture time, with the lowest MI value recorded after 3 months of culture (31.83  ±  0.81%). The highest MI value (43.77  ±  2.33%) was recorded after 3 weeks of culture; hence 3-week-old *D. caryophyllus* root segments were used in the determination of cell doubling time (Cdt).

Cell doubling time of *in vivo* grown *D. caryophyllus* root meristems was determined from 4-day-old primary root segments with a standard length of 11.15  ±  0.33 mm, while the Cdt of *in vitro* grown *D. caryophyllus* was determined from 3-week-old root segments (which demonstrated the highest MI value). The Cdt was measured by plotting the graph of the frequency of metaphase (depicted in terms of percentage) against time (duration of exposure to colchicine), which yielded linear regression lines (*y* = *mx* + *c*) for both *in vivo* and *in vitro *(Figure 1). Integration of *m* values (1.05%/hour for *in vivo* and 0.7215%/hour for *in vitro*) into Clowes' [12] formula revealed that *in vitro D. caryophyllus* had a significantly higher cell doubling time (96.07 hours) than *in vivo* plants (66.11 hours).

**Figure 2:**
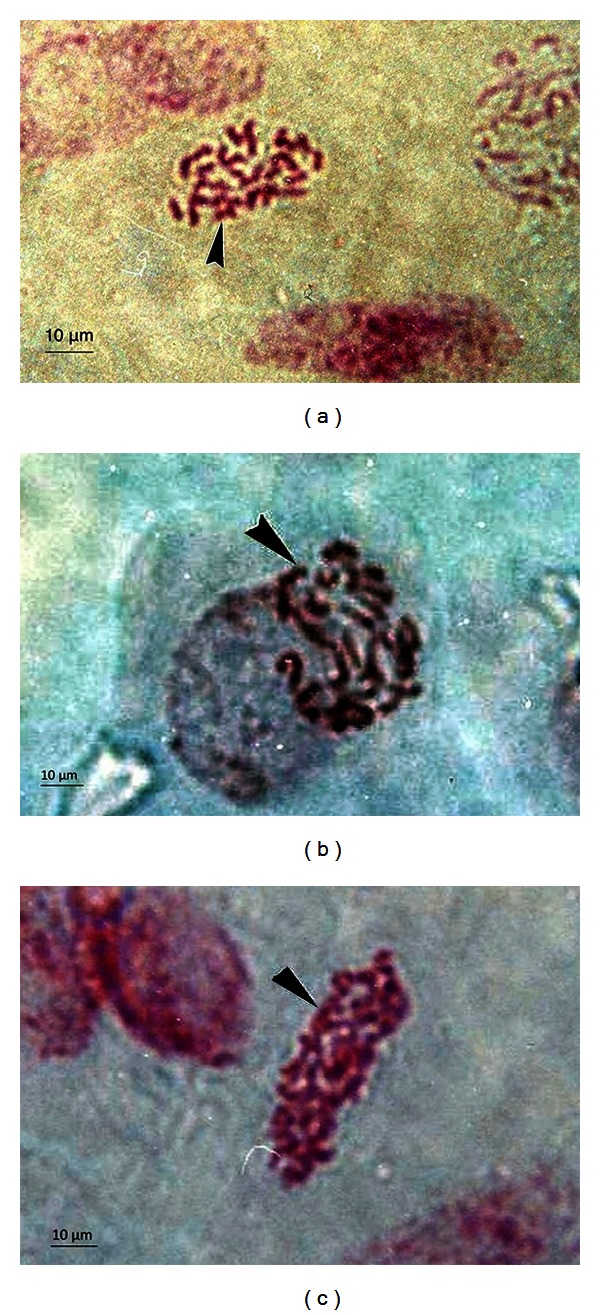
Squashed preparation of *in vitro *grown *Dianthus caryophyllus *root tip meristem; showing 30 chromosomes in a cell ((a) 1-week-old, (b) 3-month-old, and (c) 6-month-old). Arrow shows the stained chromosomes in *D. caryophyllus* meristematic cell.

On the other hand, the chromosome numbers recorded for *in vitro* grown *D. caryophyllus* showed no significant difference compared to *in vivo D. caryophyllus*, with mean chromosome numbers of 29.03 and 29.73, respectively (Table 3). It was also observed that culture time had no significant effect on the chromosome number of *in vitro* grown *D. caryophyllus *(Table 3). Feulgen-stained meristematic cells of *in vitro D. caryophyllus *at various age, showing 30 chromosomes per cell are shown in Figure 2. In contrast, the mean nuclear and cell areas of *D. caryophyllus *root meristematic cells underwent an abrupt change when transferred to *in vitro* conditions, as shown by the significant increase of both mean nuclear and cell areas after 1 day of culture (Table 4), with a mean nuclear area of 35.66  ±  0.10 *μ*m^2^ (*in vitro*) and a mean cell area of 165.05  ±  0.58 *μ*m^2^ (*in vitro*) compared to a mean nuclear area of 26.59  ±  0.09 *μ*m^2^ (*in vivo*) and a mean cell area of 142.90  ±  0.59 *μ*m^2^ (*in vivo*). However, the mean nuclear and cell areas were observed to be inconsistent and fluctuated throughout the duration of the tissue culture period (Table 4).

In general, it was found that the nuclear DNA content for both *in vivo *and *in vitro* grown *D. caryophyllus* root meristematic cells had very high percentages of polyploid cells, with a nuclear DNA C value of more than 4.8 C (Table 5). The degree of polyploid cells decreased with culture time, whereby the percentages of polyploid cells were reduced to 61.64% and 63.33% after two and three days of culture (Table 5). However, the percentage of polyploid cells was observed to be generally lower throughout the duration of the tissue culture period although the values fluctuated and appeared inconsistent (Table 5). Furthermore, it was observed that no cells were arrested at the G1 phase, except 2-week-old, 6-week-old, 7-week-old, and 4-month-old cells which showed 1.30%, 1.32%, 0.67%, and 0.68% cells arrested at the G1 phase, respectively (Table 5). It was also observed that more cells were arrested at the G2 phase compared to the G1 phase (Table 5). 

## 4. Discussion

Observations of mitotic chromosomes under the light microscope are still an informative and rapid method, essential for genomic study [5]. Changes in cell activities can be triggered when cells are transferred from one environment to a different environment [13]. For example, the transfer from *in vivo* to *in vitro* environments can trigger changes of cellular behaviour to occur [14]. Cellular behaviour of a species can be evaluated through cytological studies, such as the determination and comparison of the DNA content, chromosome count, genetic stability, and cell cycle [15]. Swartz et al. [16] stated that the transfer from *in vivo* to *in vitro* conditions can also affect the genetic constitution of the tissues. Therefore, cellular parameters such as mitotic index (MI), cell doubling time (Cdt), chromosome number, mean nuclear and cell areas, DNA content, and ploidy analysis were determined in the present investigation to elucidate any striking differences that might have occurred as a result of tissue culture procedures.

Mean mitotic indices of both *in vivo* (43.51  ±  2.14%) and *in vitro* (31.83  ±  0.81% to 43.77  ±  2.33%) grown *Dianthus caryophyllus *were quite high compared to *Petunia hybrida* [8] and *Vicia faba* [17] which showed MI values of 11.63  ±  0.26% and 13.82  ±  2.41%, respectively. Mozaffari and Gahan [18] also reported very low MI values for *Pisum sativum* (6.47  ±  1.49%), *Zea mays* (9.52  ±  1.05%), and *Allium cepa* (6.38  ±  2.18%). However, MI values differ subjected to growth conditions, for example *in vivo* or *in vitro*, and undergoing callogenesis or organogenesis. The high MI values of *D. caryophyllus *perhaps indicated that the meristematic cells were actively dividing and had a high regeneration potential. It was also observed that mean mitotic indices of *in vitro D. caryophyllus *were generally lower than in the *in vivo* grown plants, suggesting that the regeneration potential of *in vitro D. caryophyllus *decreased with culture time or the cells were adapting to culture conditions slowly. Abu Shah and Taha [19] also reported lower MI values in root cells of *in vitro *grown *Psophocarpus tetragonolobus* (3.88%) compared to *in vivo* (4.37%) plants of similar species. However, it was found that the mitotic index values fluctuated (although generally lower than *in vivo*) with culture time. A severe reduction in MI values of *D. caryophyllus *was observed after 4 days of culture, from 43.51  ±  2.14% (*in vivo*) to 32.32  ±  1.55%. The drastic reduction was probably due to tissue culture shock that occurred when the cells of *D. caryophyllus *entered the tissue culture system, resulting from the changes in growth environments from *in vivo* to *in vitro*, and could be due to the slow adaptation to the *in vitro* system. The mitotic values gradually increased until after 3 weeks of culture and reached the highest MI value (43.77  ±  2.33%) equivalent to the MI value recorded in *in vivo *plants (43.51  ±  2.14%) and subsequently decreased with culture time. This might be due to the use of growth hormones, specifically the use of auxin (NAA), which contributed to the division and cell elongation [20]. The use of growth hormones had been reported to affect the mitotic index of a species. For example, Das et al. [21] had found that the MI values of parenchyma cells of tobacco pith increased after 6 days of culture when supplemented with IAA and kinetin.

The chromosome numbers of *in vivo* and *in vitro D. caryophyllus *were similar, with approximately 30 chromosomes per cell (Table 3), in agreement with the findings by Carolin [22] in intact plants of the same species. It was also observed that the chromosomal number of *D. caryophyllus *remained stable throughout the culture period (Table 3). However, the root cells of *D. caryophyllus *failed to regenerate multiple shoots despite the chromosomal stability, probably due to the high ploidy level or increased aneuploidy level which in turn could influence the loss of organogenesis capability [23]. In the present study, it was found that the ploidy level of *D. caryophyllus *cells was high throughout the duration of the tissue culture period, with the DNA C value of more than 4.8 C (Table 5). The majority of the cells were found to be polyploid, in both *in vivo* and *in vitro* grown *D. caryophyllus*, although the percentage of polyploid cells fluctuated with culture time (Table 5). The fluctuation of the ploidy level may have an impact on the regeneration potential of the cells and tissues, for example, from 100% to 67.53% after three weeks of culture, which explained the high degree of rhizogenesis that occurred during the culture period.

Growth environments *in vitro* could possibly have enhanced the presence of more polyploid cells in *in vitro* cultured tissues due to the endoreduplication process that occurred within the population of cells, although this process can also occur *in vivo* [24]. Other factors include nuclear restitution or nuclear fragmentation caused by abnormalities such as lagging chromosomes and multipolar spindle, that often result in binucleate or multinucleate cells as well as the occurrence of aneuploidy and reduced chromosome numbers [25]. The balance of auxin and cytokinin in the culture or induction media was also reported to influence the occurrence of nuclear fragmentation and endoreduplication [25]. In the present study, no binucleate or multinucleate cells were observed; therefore it was possible that the high degree of polyploid cells in *in vivo* and *in vitro D. caryophyllus *was caused by nuclear restitution due to abnormal mitoses and chromosomal arrest at the anaphase stage [26], although not proven in the present investigation as no chromosomal aberrations had been observed. An analysis of the results showed that no somaclonal variations had occurred in *in vitro* grown* D. caryophyllus*, where both *in vivo* and *ex vitro* plants appeared morphologically similar. However, further researches are in progress to determine the effects of other growth hormones on genetic stability of *D. caryophyllus *when cultured *in vitro*.

## 5. Conclusions

Regeneration of *Dianthus caryophyllus* was successfully obtained *in vitro*. The transfer from *in vivo* to *in vitro* conditions was found to have an immediate effect on cell activity of *D. caryophyllus*, where the MI value was found to decrease, while mean cell and nuclear areas increased significantly. However, the mean cell and nuclear areas of *in vitro* grown *D. caryophyllus* appeared unstable and fluctuated throughout the 6-month culture period. Chromosome number (2*n* = 2*x* = 30) was maintained when *D. caryophyllus* entered the tissue culture system and remained stable throughout the culture period. Ploidy analysis also revealed that *in vivo* grown *D. caryophyllus* contain a high percentage of polyploid cells, which was maintained *in vitro* and throughout the 6-month culture period. Transferring the cells from *in vivo* to *in vitro* environment might have caused the already high percentage of polyploid cells to become more prominent *in vitro*.

## Figures and Tables

**Table 1:** Callus induction and rhizogenesis from root explants of *Dianthus caryophyllus* cultured on an MS medium supplemented with various hormones after 6 months of culture.

MS media + hormone (mg L^−1^)		Rhizogenesis		Callus formation (%)	Colour of callus	Observations
NAA	BAP	%	Mode			
0.0	0.0	NR	NR	NR	N/A	Necrotic

0.5	0.0	57.50^f^	Indirect	20.00^a^	White	Friable callus Roots formed after 8 days

1.0	0.0	100.00^g^	Indirect	100.00^m^	White	Friable callus Roots formed after 7 days

1.5	0.0	100.00^g^	Indirect	90.00^l^	White	Friable callus Roots formed after 7 days

2.0	0.0	100.00^g^	Indirect	100.00^m^	White	Friable callus Roots formed after 7 days

0.0	0.5	NR	N/A	NR	N/A	Necrotic after 6 weeks
	1.0	NR	N/A	NR	N/A	Necrotic after 6 weeks
	1.5	NR	N/A	NR	N/A	Necrotic after 6 weeks
	2.0	NR	N/A	NR	N/A	Necrotic after 6 weeks

0.5	0.5	5.00^a^	Indirect	35.00^d^	White	Friable callus with roots
	1.0	NR	N/A	43.00^e^	White	Friable callus with roots
	1.5	NR	N/A	NR	N/A	Necrotic after 7 weeks
	2.0	NR	N/A	25.00^b^	White	Friable callus with roots

1.0	0.5	10.00^b^	Indirect	55.00^g^	White	Friable callus with roots
	1.0	NR	N/A	82.00^j^	White	Friable callus with roots
	1.5	NR	N/A	20.00^a^	White	Friable callus with roots
	2.0	NR	N/A	NR	N/A	Necrotic after 5 weeks

1.5	0.5	NR	N/A	100.00^m^	White and green	Friable callus
	1.0	NR	N/A	75.00^h^	White and green	Friable callus
	1.5	NR	N/A	80.00^i^	White and green	Friable callus
	2.0	45.00^e^	Indirect	30.00^c^	White	Friable callus with roots

2.0	0.5	NR	N/A	75.00^h^	White and green	Friable callus
	1.0	30.00^d^	Indirect	85.00^k^	White	Friable callus with roots
	1.5	NR	N/A	50.00^f^	Green	Compact callus
	2.0	NR	N/A	30.00^c^	White and green	Friable callus

3.0	1.0	10.00^b^	Direct	NR	N/A	Necrotic after 6 weeks
	3.0	25.00^c^	Direct	30.00^c^	N/A	Friable callus with roots

*Means with different letters in the same column differ significantly at *P* < 0.05 by one-way ANOVA and Duncan's multiple range test.

(NR: no response, N/A: not available).

**Table 2:** Comparison between mitotic index (MI) of *in vivo* and *in vitro* grown *Dianthus caryophyllus* root meristems.

*Dianthus caryophyllus *	MS media + hormone (mg L^−1^)	Age	Mitotic index, MI (Percentage, %)
*In vivo *	—	4-day-old (standard growth)	43.51 ± 2.14^f^

*In vitro *	2 mg L^−1^ NAA	1-day-old	41.20 ± 0.79^e^
		2-day-old	40.23 ± 1.30^de^
		3-day-old	37.35 ± 0.40^c^
		4-day-old	32.32 ± 1.55^a^
		1-week-old	39.20 ± 1.54^cd^
		2-week-old	38.67 ± 1.35^cd^
		3-week-old	43.77 ± 2.33^f^
		4-week-old	38.62 ± 1.75^cd^
		5-week-old	38.20 ± 3.24^c^
		6-week-old	37.31 ± 1.27^c^
		7-week-old	37.69 ± 0.66^c^
		8-week-old	38.29 ± 1.87^c^
		3-month-old	31.83 ± 0.81^a^
		4-month-old	34.45 ± 0.70^b^
		6-month-old	33.17 ± 0.78^ab^

*Means with different letters in the same column differ significantly at *P* < 0.05 by one-way ANOVA and Duncan's multiple range test.

**Table 3:** Comparison between chromosome numbers of *in vivo* and *in vitro* grown *Dianthus caryophyllus* root meristems.

*Dianthus caryophyllus *	MS media + hormone (mg L^−1^)	Age	Chromosome number (mean)
*In vivo *	—	4-day-old (standard growth)	29.73 ± 0.12^a^

*In vitro *	2 mg L^−1^ NAA	1-day-old	29.40 ± 0.31^a^
		2-day-old	28.87 ± 0.29^a^
		3-day-old	29.67 ± 0.16^a^
		4-day-old	29.00 ± 0.48^a^
		1-week-old	29.47 ± 0.40^a^
		2-week-old	29.87 ± 0.24^a^
		3-week-old	29.93 ± 0.42^a^
		4-week-old	29.53 ± 0.51^a^
		5-week-old	28.80 ± 0.39^a^
		6-week-old	28.73 ± 0.44^a^
		7-week-old	28.67 ± 0.36^a^
		8-week-old	27.93 ± 0.43^a^
		3-month-old	28.27 ± 0.60^a^
		4-month-old	28.31 ± 0.69^a^
		6-month-old	29.00 ± 0.60^a^

*Means with different letters in the same column differ significantly at *P* < 0.05 by one-way ANOVA and Duncan's multiple range test.

**Table 4:** Comparison between mean nuclear and cell areas of *in vivo* and *in vitro* grown *Dianthus caryophyllus* root meristems.

*Dianthus caryophyllus *	MS media + hormone (mg L^−1^)	Age	Mean nuclear area, *μ*m^2^ (N)	Mean cell area, *μ*m^2^ (C)	Ratio (N/C)
*In vivo *	—	4-day-old (standard growth)	26.59 ± 0.09^bcd^	142.90 ± 0.59^def^	0.19^a^

*In vitro *	2 mg L^−1^ NAA	1-day-old	35.66 ± 0.10^efgh^	165.05 ± 0.58^g^	0.22^ab^
		2-day-old	30.34 ± 0.09^bcdef^	124.73 ± 0.44^c^	0.24^abc^
		3-day-old	42.37 ± 0.12^hi^	153.06 ± 0.48^fg^	0.28^cd^
		4-day-old	32.30 ± 0.08^cdefg^	118.94 ± 0.04^bc^	0.27^bcd^
		1-week-old	43.95 ± 0.28^i^	181.48 ± 0.49^h^	0.24^abc^
		2-week-old	29.64 ± 0.11^bcde^	145.58 ± 0.56^ef^	0.20^a^
		3-week-old	17.66 ± 0.06^a^	83.27 ± 0.31^a^	0.21^a^
		4-week-old	33.03 ± 0.10^defg^	106.57 ± 0.30^b^	0.31^d^
		5-week-old	30.48 ± 0.11^bcdef^	129.92 ± 0.54^cd^	0.23^abc^
		6-week-old	25.95 ± 0.09^bc^	131.28 ± 0.56^cde^	0.20^a^
		7-week-old	38.21 ± 0.12^ghi^	142.23 ± 0.59^def^	0.27^bcd^
		8-week-old	30.23 ± 0.10^bcdef^	143.38 ± 0.54^def^	0.21^a^
		3-month-old	36.50 ± 0.12^efgh^	157.14 ± 0.60^fg^	0.23^abc^
		4-month-old	36.88 ± 0.12^fgh^	153.14 ± 0.61^fg^	0.24^abc^
		6-month-old	24.53 ± 0.09^b^	105.27 ± 0.44^b^	0.23^abc^

*Means with different letters in the same column differ significantly at *P* < 0.05 by one way ANOVA and Duncan's multiple range test.

**Table 5:** Comparison of percentage of nuclei in various cell cycle phases between *in vivo* and *in vitro* grown *Dianthus caryophyllus* root meristems.

*Dianthus caryophyllus *	MS media + hormone (mg L^−1^)	Age	Cell cycle phase (%)			Polyploidy (%)
			G1	S	G2	
*In vivo *	—	4-day-old (standard growth)	—	1.32^a^	3.95^a^	94.74^ij^

*In vitro *	2 mg L^−1^ NAA	1-day-old	—	—	—	100.00^j^
		2-day-old	—	11.32^f^	27.04^g^	61.64^bc^
		3-day-old	—	0.67^a^	36.00^h^	63.33^bcd^
		4-day-old	—	3.27^bc^	9.15^b^	87.58^ghi^
		1-week-old	—	2.01^ab^	22.82^f^	75.17^ef^
		2-week-old	1.30^b^	17.63^i^	20.78^de^	60.39^b^
		3-week-old	—	11.04^f^	21.43^ef^	67.53^bcde^
		4-week-old	—	1.42^a^	3.55^a^	95.04^ij^
		5-week-old	—	9.09^e^	19.58^d^	71.33^de^
		6-week-old	1.32^b^	3.97^c^	9.27^b^	85.43^gh^
		7-week-old	0.67^a^	12.67^g^	20.67^de^	66.00^bcde^
		8-week-old	—	14.00^h^	43.33^i^	42.67^a^
		3-month-old	—	6.08^d^	13.51^c^	80.41^fg^
		4-month-old	0.68^a^	0.68^a^	4.73^a^	93.92^hij^
		6-month-old	—	18.75^i^	10.42^b^	70.83^cde^

*Means with different letters in the same column differ significantly at *P* < 0.05 by one way ANOVA and Duncan's multiple range test.

(—: absent/no data observed).

## Abbreviations

BAP: 6-Benzyl aminopurine
NAA: *α*-Naphthalene acetic acid.
